# Modification and Applicability of Questionnaires to Assess the Recovery-Stress State Among Adolescent and Child Athletes

**DOI:** 10.3389/fphys.2019.01414

**Published:** 2019-11-20

**Authors:** Sarah Kölling, Alexander Ferrauti, Tim Meyer, Mark Pfeiffer, Michael Kellmann

**Affiliations:** ^1^Faculty of Sport Science, Ruhr University Bochum, Bochum, Germany; ^2^Department of Sport Science, Stellenbosch University, Stellenbosch, South Africa; ^3^Institute of Sports and Preventive Medicine, Saarland University, Saarbrücken, Germany; ^4^Institute of Sports Science, Johannes Gutenberg University Mainz, Mainz, Germany; ^5^School of Human Movement and Nutrition Sciences, The University of Queensland, St Lucia, QLD, Australia

**Keywords:** training, monitoring, psychometrics, development, sports

## Abstract

Despite the general consensus regarding the implementation of self-report measures in the training monitoring, there is a lack of research about their applicability and comprehensibility among developing athletes. However, this target group needs special considerations to manage the increasing training demands while maintaining health and performance. This study deals with challenges of applying recovery-stress questionnaires which were validated with adult populations among developing athletes and presents a possible approach to enhance their applicability. In two phases, the Acute Recovery and Stress Scale (ARSS), a 32-adjective list covering eight scales, and the 8-item derived version, the Short Recovery and Stress Scale (SRSS) were answered by 1052 athletes between 10 and 16 years. Phase 1 included 302 14- to 16-year-old athletes who used the original questionnaires with the additional option to mark “*I don’t understand*,” while modified versions with additional explanations (phase 2) were applied to 438 adolescents (14.7 ± 0.6 years) and 312 child athletes (11.8 ± 1.1 years). Data of the original validation sample (*n* = 442) were reanalyzed to examine measurement invariance between adults and adolescents. The results showed comparable psychometric properties to the validation sample (e.g., *r*_*it*_ > 0.30) and acceptable fit indices via confirmatory factor analyses (CFA), although more difficulties and limitations were present within the younger groups (e.g., Cronbach’s α between 0.50 and 0.87), especially among 10- and 11-year-olds. The original as well as the modified SRSS, on the other hand, indicated good applicability (Cronbach’s α between 0.72 and 0.80). Multigroup CFA revealed measurement invariance of the original ARSS among adults and adolescents and of the modified ARSS among adolescents and children. Overall, the present study confirmed the assumption that questionnaires designed by and for adults cannot be directly transferred to younger athletes. The peculiarities and differences in the cognitive and affective development of each age group need to be considered. Future research needs to identify a cut-off age to start the proper use of psychometric tools, especially for state-oriented assessments for routine application in training monitoring. Further modifications and long-term investigations are necessary to implement psychometric monitoring in high-performance environments within youth sport.

## Introduction

Despite the goal of the International Olympic Committee to develop healthy, capable, and resilient young athletes (Bergeron et al., 2015), training demands on developing athletes are high in order to achieve the elite level. In addition to their sport, these athletes are facing a double burden due to school and social commitments and other non-sport stressors. Life event stress, as an example, was shown to predict injury occurrence among junior soccer players (Johnson and Ivarsson, 2011). In general, there is consensus about the necessity to manage an adequate balance between stress and recovery (Kellmann and Beckmann, 2018; Kellmann et al., 2018), which is supported by the systematic review of Drew and Finch (2016) who are indicating an emerging moderate evidence for the relationship between training load and the risk of injury and illness. Therefore, effective management of training and competition, such as periodization or the length of mid-season and off-season breaks, plays an essential role in the maintenance of performance and injury prevention (Jones et al., 2017). For instance, Phibbs et al. (2018) recently analyzed the weekly match and training loads of adolescent rugby union players during 14 weeks. They found a large within-player variability that represented the inconsistent match scheduling which, furthermore, exposed the players to an increased risk of injury. According to a recent systematic review of longitudinal studies investigating the association between training load with injury and illness, it is not only the magnitude of external training load but also the increase of the intensity of external load (e.g., speed, weights) as well as the internal load (e.g., perceived exertion, heart rate) which result in an augmented stress and injury risk (Jones et al., 2017). Excessive training overload combined with inadequate recovery may lead to non-functional overreaching (NFOR) and can develop into the overtraining syndrome (OTS) which is characterized by symptoms of fatigue, performance decline, and mood disturbances (Meeusen et al., 2013).

DiFiori et al. (2014) raise the concern of overuse injury and burnout resulting from an increased pressure to begin with high-intensity training and the emphasis on competitive success already in youth sport. In their position statement, they point out the lack of research on the incidence and prevalence of overuse injuries in children and adolescents. Nevertheless, there is some evidence supporting the relevance and need for special attention to develop prevention programmes. A survey among 11- to 18-year-old English athletes (*N* = 376) revealed that approximately one third has experienced a state of NFOR or OTS (Matos et al., 2011). Similar rates were found in adolescent swimmers (*N* = 231) across Greece, Japan, Sweden, and the United States, with 34.6% of the total sample and a range from 20.5 to 45.1% between countries (Raglin et al., 2000). Bergeron et al. (2015) emphasize that there is still a lack of evidence-based injury prevention strategies in sports with a high risk of injury, such as rugby, field hockey, soccer, volleyball, running, lacrosse, gymnastics, martial arts, tennis, and wrestling.

One important approach is monitoring the athlete’s training response and recovery-stress state to ensure the readiness to perform as well as to sustain the athlete’s health and well-being and prevent injuries in the long-term (Murray, 2017; Kellmann et al., 2018). This is further important in terms of effective talent development and preserving the limited talent pool (Murray, 2017). Especially among adolescent athletes, it seems important to take into account their individual perception and assessment of the training load, as Brink et al. (2014) have shown that under-17 and under-19 soccer players perceived the training as harder than it was intended to be by the coach. Even though coaches showed an altered rating of observed exertion to align with the athletes’ responses after training sessions, small to moderate differences were still found in a study of youth hockey, netball, rugby, and soccer players (Scantlebury et al., 2018). Despite the documentation of the training load and measuring the internal load via physiological responses (e.g., heart rate, creatine kinase), self-report measures are a vital source of information (Kellmann, 2000; Bourdon et al., 2017; Scantlebury et al., 2017). As the manifestation of the OTS is a process over a period of time, psychological changes and mood disturbances have been identified as successful indicators (Steinacker et al., 1999; Meeusen et al., 2013). According to a systematic review, acute and chronic training loads were better reflected by subjective measures indicating an impaired well-being following acute increases of training as well as chronic training and improvements after acute decreases in training load (Saw et al., 2016). Considering the implementation of psychometric monitoring tools, Saw et al. (2017) highlight the importance of established questionnaires which fulfill the quality criteria in terms of a theoretical basis, reliability, and validity. While there is a number of instruments available (for an overview see Nässi et al., 2017b), their applicability among adolescents or even children needs to be considered critically and should not be applied before thorough pretesting (Borgers et al., 2000). While it seems that, with the help of parents, children at the age of five may already be able to provide reliable and valid replies to their health-related quality of life (Varni et al., 2007), Williams et al. (1994) point out that young people may have difficulties applying the *Rating of Perceived Exertion* scale (Borg, 1998), as it demands comprehension and translation of the verbal expressions and the range of numbers to their presumably rudimentary concept of exercise and the accompanied sensations. Therefore, the *Children’s Effort Rating Table* has been developed for 6- to 9-year old children (Williams et al., 1994). Another modification has been reported by Yelling et al. (2002) who have illustrated the verbal and numerical rating scale with pictorial images of exertion. However, the recovery-stress continuum is multi-dimensional and cannot be simplified by assessing only the exertion or the absence thereof (Kellmann, 2010; Heidari et al., 2018). While it is recommended to capture different aspects of recovery and stress (e.g., mood, emotional well-being), it is doubtful whether existing questionnaires which were developed and validated among adults can be transferred to be used on younger athletes. In general, there are two requirements that need to be fulfilled before implementing self-report measures in this context, i.e., the cognitive development to read and understand the items and the children’s level of self-perception to differentiate their current psychophysiological state and its representation on rating scales. Borgers et al. (2000) differentiate between reading ability, which involves the vocabulary in general and its decoding, and language ability which involves reading comprehension.

An eligible tool for training monitoring is the Acute Recovery and Stress Scale (ARSS) and its shortened version, the Short Recovery and Stress Scale (SRSS, Kellmann et al., 2016) which are established instruments to assess multiple facets of recovery and stress states (i.e., physical, mental, emotional, and overall dimensions). These were developed to support every-day and long-term training monitoring by showing sensitivity to change in an economical way (Hitzschke et al., 2017). Several studies indicate their sport-specific applicability as well as validity in different training settings (Kölling et al., 2015; Collette et al., 2018; Pelka et al., 2018). However, their application for athletes younger than 16 years has not been examined yet. As the 32 items of the ARSS assess the current recovery-stress state on the basis of single adjectives, the understanding of them by children and adolescents needs to be investigated. A particularity of the SRSS is its derivation of the ARSS’s scales. While four items are comprised into one of the ARSS’s scales, these eight scales are assessed as single items in the SRSS and represent a somewhat broader construct of the recovery-stress dimensions. The corresponding adjectives (ARSS items) serve as descriptors below each SRSS item to support their meaning. However, it needs to be verified whether additional explanations are needed among younger athletes. The present study aims at pointing out likely challenges of application and demonstrating possible approaches to modify and adapt existing tools for younger athletes.

## Materials and Methods

### Participants

Overall, 1052 athletes (75.6% male) participated in the different phases of the study. The majority (83.9%) was engaged in team sports such as soccer and handball, while 15.8% belonged to individual sports. Table 1 provides an overview of participants’ characteristics in each of the phases. The group of phase 1 consisted of 302 athletes between 14 and 16 years. Most of the data was collected in several selection-focused training camps. During a nationwide selection course of the handball association, 239 players of that age group were recruited. Additionally, 17 athletes were part of an under-15 and 21 athletes of an under-16 soccer team. In order to retain the anonymity of the athletes and to prevent distorted responses, the questionnaires were answered without individual demographic information. In phase 2, participants were divided into the group of adolescents between 14 and 16 years (*n* = 438) and child athletes between 10 and 13 years (*n* = 312). The athletes and their parents were informed about the purpose of the study and informed consent was attained by athletes as well as parents prior to the data collection. Ethical approval was obtained by the local ethic committee.

**TABLE 1:** Overview of the studies, participants’ characteristics and response patterns.

	**Reference Sample^a^**	**Phase 1**	**Phase 2**	**Phase 2**
Age group	Adults (≥16 years)	Adolescents (14–16 years)	Adolescents (14–16 years)	Children (10–13 years)
Questionnaires	Original ARSS, Original SRSS	Original ARSS + “*I don’t understand*,” Original SRSS	Modified ARSS + “*I don’t understand*,” Modified SRSS	Modified ARSS + “*I don’t understand*,” Modified SRSS
*N* (male, female)	574 (279, 293)	302 (183, 119)	438 (383, 55)	312 (232, 79)
Age (*M* ± *SD*)	21.0 ± 6.8	14–16	14.7 ± 0.6	11.8 ± 1.1
Complete item responses (*n* [%])		202 (66.9%)	263 (60.0%)	118 (37.8%)
Percentage of item non-responses		0.8%	1.1%	1.5%
Percentage of “*I don’t understand*” responses		0.8%	0.7%	4.5%

*ARSS, Acute Recovery and Stress Scale; SRSS, Short Recovery and Stress Scale; ^*a*^, reference values as published in the German manual (Kellmann et al., 2016, p. 27).*

### Procedure

The study consisted of two evaluation phases which were conducted successively (Table 1). In phase 1, the ARSS was applied among adolescents with the option to mark “*I don’t understand*” beside the original rating scale, while the SRSS remained in its original form. Following initial feedback based on the answers and the most common ratings, four items were identified and modified with additional adjectives to test them in phase 2 among another group of adolescents and child athletes. As a second alteration, the SRSS was also modified with a sentence for each item to describe the different domains of recovery and stress. In each phase of data collection, the questionnaires were answered in a paper version. As the questionnaires were distributed among cooperating sports clubs, the researchers were not present during the process of completing them. The athletes were instructed by the persons who handed out the scales. As the psychometric parameters of the study will be compared with statistics of the original (e.g., dispersion measures, correlation coefficients, Cronbach’s alpha, fit indices), the characteristics of the validation sample which were presented in the manual serve as reference values (Table 1).

## Instruments

The ARSS is a 32-item adjective list (e.g., “*rested*,” “*tired*”) that is rated from 0 (“*does not apply at all*”) to 6 (“*fully applies*”) (Kellmann et al., 2016; Kellmann and Kölling, 2019). Eight scales are then generated by summarizing four items which cover the *Recovery* dimension (*Physical Performance Capability*, *Mental Performance Capability*, *Emotional Balance*, *Overall Recovery*) and the *Stress* dimension (*Muscular Stress*, *Lack of Activation*, *Negative Emotional State*, *Overall Stress*). As depicted in Table 2, the original ARSS showed satisfactory discriminatory power of the items (*r*_*it*_ = 0.51 to 0.82) and, as shown in Table 3, good scale homogeneity (α = 0.76 to 0.90) for the validation sample (*N* = 574, 21 ± 6.8 years). The factorial structure of the original was further supported via confirmatory factor analysis (Kellmann et al., 2016).

**TABLE 2:** Means, standard deviations and item-total correlations of the ARSS.

		**Reference Values^a^: Adults (*N* = 574)**			**Phase 1: Adolescents (*n* = 202)**			**Phase 2: Adolescents (*n* = 263)**			**Phase 2: Children (*n* = 118)**		
		**M**	**SD**	***r*_it_**	**M**	**SD**	***r*_it_**	**M**	**SD**	***r*_it_**	**M**	**SD**	***r*_it_**
**Recovery**	**Physical Performance Capability**												
**Dimension**	Item 1	3.4	1.4	0.77	4.4	1.1	0.68	4.1	1.3	0.72	4.4	1.4	0.53
	Item 2	4.0	1.4	0.71	4.8	1.1	0.59	4.7	1.2	0.70	5.1	1.1	0.47
	Item 3	3.3	1.5	0.79	4.2	1.3	0.69	4.1	1.4	0.75	4.6	1.3	0.69
	Item 4	3.3	1.5	0.82	4.3	1.3	0.75	4.2	1.5	0.72	4.7	1.3	0.70
	**Mental Performance Capability**												
	Item 1	4.0	1.3	0.59	4.9	1.06	0.63	4.6	1.2	0.73	4.6	1.3	0.57
	Item 2	4.2	1.3	0.67	5.0	1.13	0.52	4.6	1.3	0.66	4.9	1.2	0.43
	Item 3	3.9	1.3	0.74	4.7	1.08	0.62	4.7	1.2	0.67	4.8	1.2	0.59
	Item 4	3.5	1.4	0.68	4.6	1.12	0.69	4.5	1.2	0.59	4.6	1.4	0.50
	**Emotional Balance**												
	Item 1	4.0	1.5	0.55	4.4	1.1	0.36	4.6	1.3	0.53	5.1	1.1	0.40
	*Item 2*	3.5	1.4	0.51	4.2	1.4	0.31	4.1	1.4	0.55	4.2	1.4	0.18
	Item 3	4.2	1.4	0.60	5.3	1.0	0.46	4.9	1.2	0.60	5.2	1.0	0.34
	Item 4	3.7	1.4	0.58	4.6	1.2	0.38	4.3	1.3	0.51	4.5	1.3	0.31
	**Overall Recovery**												
	Item 1	3.4	1.4	0.70	4.0	1.2	0.66	3.8	1.4	0.66	4.2	1.5	0.57
	Item 2	3.0	1.5	0.72	3.8	1.4	0.64	3.6	1.6	0.66	3.8	1.7	0.61
	Item 3	2.9	1.5	0.65	4.0	1.4	0.52	3.5	1.6	0.66	4.0	1.8	0.43
	Item 4	3.0	1.5	0.70	4.0	1.4	0.71	3.7	1.5	0.66	4.2	1.7	0.63
**Stress**	**Muscular Stress**												
**Dimension**	Item 1	2.3	1.6	0.74	1.3	1.3	0.67	1.8	1.5	0.68	1.3	1.7	0.65
	Item 2	2.6	1.7	0.77	1.5	1.4	0.68	1.9	1.6	0.73	1.1	1.4	0.67
	*Item 3*	1.8	1.6	0.75	1.1	1.3	0.66	1.9	1.7	0.64	1.3	1.8	0.64
	Item 4	2.5	1.8	0.66	1.3	1.4	0.67	1.8	1.6	0.60	1.5	1.7	0.53
	**Lack of Activation**												
	Item 1	1.6	1.6	0.70	0.5	0.9	0.39	0.8	1.3	0.66	0.6	1.2	0.57
	Item 2	1.6	1.6	0.74	0.7	1.1	0.56	1.1	1.4	0.66	0.7	1.4	0.53
	Item 3	1.6	1.6	0.71	0.3	0.8	0.52	0.7	1.2	0.70	0.5	1.2	0.63
	Item 4	2.0	1.6	0.65	0.9	1.1	0.55	1.2	1.4	0.53	0.6	1.2	0.46
	**Negative Emotional State**												
	*Item 1*	1.8	1.7	0.59	0.7	1.1	0.34	1.0	1.4	0.53	0.7	1.3	0.51
	Item 2	2.2	1.7	0.56	0.9	1.1	0.53	1.4	1.4	0.55	1.1	1.3	0.54
	Item 3	1.7	1.6	0.66	0.5	0.9	0.61	0.9	1.3	0.69	0.8	1.3	0.54
	Item 4	2.1	1.7	0.61	1.0	1.4	0.51	1.6	1.6	0.45	1.5	1.7	0.36
	**Overall Stress**												
	*Item 1*	2.8	1.7	0.71	1.6	1.4	0.58	2.0	1.6	0.67	1.6	1.8	0.58
	Item 2	2.1	1.7	0.76	1.3	1.3	0.64	1.7	1.5	0.76	1.4	1.6	0.71
	Item 3	2.0	1.6	0.70	1.0	1.2	0.66	1.6	1.5	0.70	1.0	1.4	0.60
	Item 4	2.5	1.8	0.76	1.2	1.3	0.64	1.8	1.7	0.75	1.1	1.5	0.66

*ARSS, Acute Recovery and Stress Scale; ^*a*^, reference values as published in the German manual (Kellmann et al., 2016, p. 41); items in italic font indicate modification in phase 2.*

**TABLE 3:** Values of the internal consistency (Cronbach’s α) of the ARSS scales across the groups.

		**Reference Values^a^: Adults (*N* = 574)**	**Phase 1: Adolescents (*n* = 202)**	**Phase 2: Adolescents (*n* = 263)**	**Phase 2: Children (*n* = 118)**
Recovery Dimension	Physical Performance Capability	0.90	0.84	0.87	0.78
	Mental Performance Capability	0.84	0.80	0.83	0.73
	*Emotional Balance*	0.76	0.59	0.75	0.50
	Overall Recovery	0.85	0.81	0.83	0.76
Stress Dimension	*Muscular Stress*	0.87	0.84	0.83	0.80
	Lack of Activation	0.86	0.71	0.81	0.75
	*Negative Emotional State*	0.79	0.70	0.75	0.69
	*Overall Stress*	0.88	0.81	0.87	0.81

*ARSS, Acute Recovery and Stress Scale; ^*a*^, reference values as published in the German manual (Kellmann et al., 2016, p. 41); scales in italic font contained one modified item in phase 2.*

The SRSS is a derivation of the ARSS using the eight scales as items which are rated on the scale from 0 to 6 (Kellmann et al., 2016; Kellmann and Kölling, 2019). The four related adjectives are listed as descriptors of the items to provide examples of each construct. The *Short Recovery Scale* (*Physical Performance Capability*, *Mental Performance Capability*, *Emotional Balance*, and *Overall Recovery*) and the *Short Stress Scale* (*Muscular Stress*, *Lack of Activation*, *Negative Emotional State*, and *Overall Stress*) revealed acceptable discriminatory power (*r*_*it*_ = 0.37 to 0.66) as well as satisfactory scale homogeneity with α = 0.70 and 0.76, respectively, in the validation sample (Tables 4, 5, respectively).

**TABLE 4:** Means, standard deviations and item-total correlations of the SRSS.

		**Reference Values^a^: Adults (*N* = 574)**			**Phase 1: Adolescents (*n* = 199)**			**Phase 2: Adolescents (*n* = 261)**			**Phase 2: Children (*n* = 115)**		
		**M**	**SD**	***r*_*it*_**	**M**	**SD**	***r*_*it*_**	**M**	**SD**	***r*_*it*_**	**M**	**SD**	***r*_*it*_**
Recovery	PPC	4.2	1.5	0.62	4.4	1.1	0.64	4.4	1.2	0.63	4.9	1.1	0.46
Dimension	MPC	4.4	1.3	0.51	4.7	1.0	0.54	4.7	1.1	0.60	4.9	1.0	0.61
	EB	4.3	1.5	0.37	4.7	1.0	0.47	4.6	1.1	0.48	5.0	1.0	0.42
	OR	3.7	1.6	0.53	4.1	1.3	0.61	3.9	1.4	0.63	4.4	1.3	0.60
Stress	MS	3.1	1.8	0.49	1.6	1.4	0.58	2.3	1.6	0.61	1.6	1.8	0.67
Dimension	LA	2.4	1.9	0.58	0.5	0.9	0.47	1.1	1.4	0.62	0.9	1.4	0.43
	NES	2.4	1.9	0.48	0.9	1.3	0.39	1.2	1.4	0.55	0.9	1.2	0.45
	OS	2.9	1.8	0.66	1.4	1.3	0.63	1.9	1.6	0.70	1.5	1.6	0.58

*SRSS, Short Recovery and Stress Scale; PPC, Physical Performance Capability; MPC, Mental Performance Capability; EB, Emotional Balance; OR, Overall Recovery; MS, Muscular Stress; LA, Lack of Activation; NES, Negative Emotional State; OS, Overall Stress; ^*a*^, reference values as published in the German manual (Kellmann et al., 2016, p. 54).*

**TABLE 5:** Values of the internal consistency (Cronbach’s α) of the *Short Recovery Scale* and the *Short Stress Scale* across the groups.

	**Reference Values^a^: Adults (*N* = 574)**	**Phase 1: Adolescents (*n* = 199)**	**Phase 2: Adolescents (*n* = 261)**	**Phase 2: Children (*n* = 115)**
Short Recovery Scale	0.70	0.76	0.78	0.73
Short Stress Scale	0.76	0.72	0.80	0.73

*^*a*^, reference values as published in the German manual (Kellmann et al., 2016, p. 54).*

In phase 1 of the study, each item of the ARSS could also be answered with the option “*I don’t understand*” next to the Likert-type rating scale, while the original SRSS was used.

In phase 2, one ARSS item each of four scales (i.e., *Emotional Balance*, *Muscular Stress*, *Negative Emotional State*, and *Overall Stress*) was modified with additional descriptions. These were added in brackets behind each item [e.g., “*depressed* (e.g., *feeling down*)”]. Additionally, “*I don’t understand*” (next to the rating scale) could be ticked as well. For the SRSS, a sentence was added to each item (e.g., *Physical Performance Capability*: “*I am full of energy and feel ready for training/competition*”).

### Statistical Analyses

In this publication, three statistical approaches were examined. The first step was a descriptive analysis using SPSS 25 to compare means and standard deviations separated by the different groups in each phase (i.e., adolescents and children). For single items, discriminatory power was assessed via corrected item-total correlations (*r*_*it*_). Cronbach’s α was determined to analyze internal consistency of the scales. In addition, response patterns of each group were analyzed and the “*I don’t understand*” responses are displayed divided into the single age subgroups. Due to the missing demographic information in phase 1, the frequency of these responses (in percentage) is presented only for the participants of phase 2. Spearman correlation coefficients (*r*_*s*_) were calculated to examine the relationship between the ARSS scales and the corresponding SRSS items. The descriptive values which were reported in the manual serve as benchmark for the present study.

The second approach was to perform confirmatory factor analyses (CFA) and, as a third approach, to examine measurement invariance of the ARSS using R (Lavaan package version 0.6-3 by Rosseel, 2012; semTools package version 0.5-1 by Jorgensen et al., 2018). For this purpose, parts of the original data set of the validation sample was reanalyzed and fit indices were compared with the adolescent sample of phase 1. Only data of participants above 16 years were used from the validation sample to avoid an overlap of that age category. This reduced the sample size to *n* = 442 among the adults. Separate CFA’s were performed among children and adolescents of the current data collection, as a modified questionnaire was used in phase 2. For the default model, inferential and descriptive fit statistics and the critical thresholds were selected [i.e., χ^2^ with df and *p*-values, comparative fit index (CFI > 0.90), root mean square error of approximation (RMSEA < 0.08) ± 90% confidence interval [90%-CI], standardized root mean residual (SRMR < 0.10)] as commonly reported in the literature (Hu and Bentler, 1999; Beauducel and Wittmann, 2005). Robust maximum likelihood estimators were applied to account for non-normal multivariate distribution. To examine measurement invariance across groups, i.e., if the recovery and stress models are comparable between the samples, multigroup CFA was conducted (Cheung and Rensvold, 2002). In a first step, the least restrictive model was estimated to analyze the same associations of items and factors, and the same number of factors (i.e., configural invariance). For the second model, all factor loadings were constrained to be invariant across groups to analyze metric invariance (i.e., weak measurement invariance). A third model tested whether the observed indicators show equal intercepts when regressed on the latent factors (i.e., scalar/strong invariance). Change of the fit indices were evaluated based on recommendations by Cheung and Rensvold (2002) for CFI (i.e., ΔCFI ≤ −0.01) and by Chen (2007) for changes of RMSEA (i.e., ΔRMSEA < 0.015) and SRMR (i.e., ΔSRMR < 0.01), whereas χ^2^-Difference test was not performed as both references do not recommend it and as the test provided by the semTools package is not applicable to the robust estimation method.

Due to the exploratory nature of the study, statistical analyses were performed only with those participants who provided complete responses. As a consequence, the sample sizes were reduced considerably for all groups (i.e., adolescents phase 1: *n* = 202, adolescents phase 2: *n* = 263, children phase 2: *n* = 118).

## Results

**FIGURE 1:**
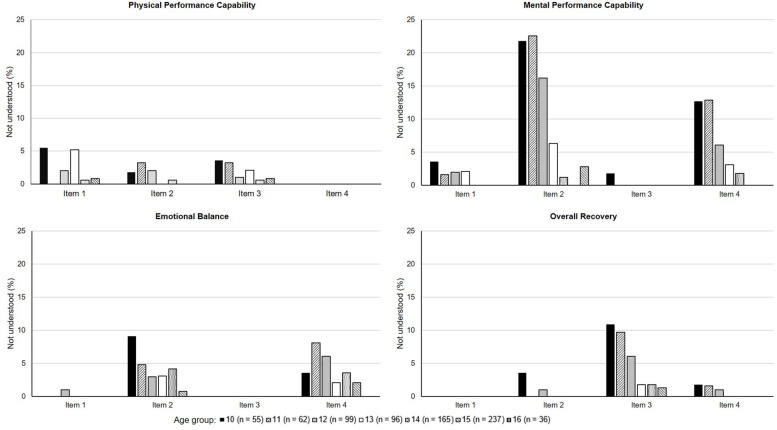
Percentages of items that were not understood within the *Recovery* dimension separated by age subgroups of phase 2.

**FIGURE 2:**
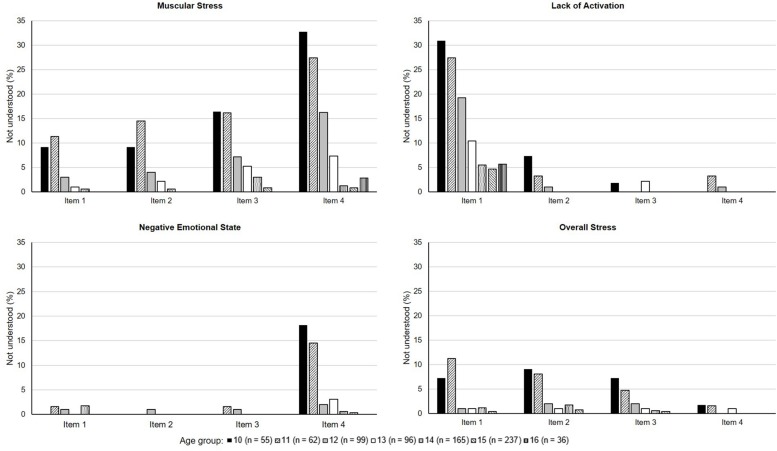
Percentages of items that were not understood within the *Stress* dimension separated by age subgroups of phase 2.

Response rates are depicted in Table 1. The children group provided the majority of missing data with 37.5% rating the ARSS items completely, while 4.5% of missing values were attributable to the “*I don’t understand*” rating. Up to two thirds (phase 1) and more than half (phase 2) of the adolescent groups returned fully completed ARSS ratings, respectively. Less than 1% of missing data was accounted for by “*I don’t understand*” answers. Figure 1 shows the percentages of items from the *Recovery* dimension which the participants of phase 2 answered with “*I don’t understand*.” Figure 2 displays the percentages for the *Stress* dimension. Within the *Recovery* dimension, there was no item that was not understood by more than 20% of each age group, with the exception of one item in the scale *Mental Performance Capability* which the 10- (21.8%) and 11-year-olds (22.6%) did not understand. Within the *Stress* dimension, over 30% of the 10- and 27.4% of the 11-year-olds marked the same items of *Muscular Stress* and *Lack of Activation* as difficult to understand.

Table 2 shows means, standard deviations, and item-total correlations for the three groups of the study compared to the original data of the validation sample as reported in the manual (Kellmann et al., 2016). On the descriptive level, all of the *Recovery* scores were higher than the original data. Among the *Stress* dimension, scores of each group were apparently lower than in the validation sample. Values were rarely >2. The standard deviations, on the other hand, appeared somewhat similar across the different groups. Item-total correlations ranged within comparable degrees between the groups. In the children group of phase 2, discriminatory power was rather weak (i.e., *r*_*it*_ = 0.18) for just one of the items that had been modified with an explanation. The remaining coefficients reached values above 0.30 across the different groups. Table 3 compares the Cronbach’s α values of the three groups with the original data of the validation sample. As these analyses were performed with complete responses, participants who marked “*I don’t understand*” were not included. The validation sample of the original population presented the highest values throughout the scales, while the lowest values were found among the child athletes. *Emotional Balance*, in particular, revealed poor internal consistency (α = 0.50), while the remaining scales showed acceptable ranges of Cronbach’s α. Among adolescents, however, increased values can be identified when comparing phase 1 and phase 2, where the scale contained one modified item (i.e., α = 0.59 vs. α = 0.75). Improved values were also identified for *Negative Emotional State* (i.e., α = 0.70 vs. α = 0.75) and for *Overall Stress* (i.e., α = 0.81 vs. α = 0.87).

Table 4 provides an overview of the SRSS’s means, standard deviations and item-total correlations for the three groups and the validation sample. While means of the *Short Recovery Scale* appeared to be similar across the different samples, the validation sample presented higher scores among the *Short Stress Scale* compared to the study groups. Item-total correlations were above 0.30 across all groups. A comparison of Cronbach’s α values of the *Short Recovery Scale* and the *Short Stress Scale* can be found in Table 5. For all groups, the *Short Recovery Scale* showed higher internal consistency than the validation sample, while Cronbach’s α of the *Short Stress Scale* was higher in the validation sample compared to phase 1 adolescents and phase 2 children.

Spearman correlations between the ARSS scales and the corresponding SRSS items are shown in Table 6. Compared to the validation sample, similar or higher relationships within the *Recovery* dimension were identified across the three study groups. Within the *Stress* dimension, correlation coefficients were higher in the validation sample, whereas phase 2 adolescents revealed the highest correlation among *Overall Stress* of all groups. Strong correlations (i.e., *r*_*s*_ ≥ 0.70) appeared only within the validation sample (*Lack of Activation*, *Negative Emotional State*) and within adolescents in phase 2 (*Physical Performance Capability*, *Overall Recovery*, *Overall Stress*).

**TABLE 6:** Spearman correlations between the ARSS scales and corresponding SRSS items across the groups.

	**Reference Values^a^: Adults**	**Phase 1: Adolescents**	**Phase 2: Adolescents**	**Phase 2: Children**
	**(*N* = 574)**	**(*n* = 199)**	**(*n* = 261)**	**(*n* = 115)**
Physical Performance Capability	0.62	0.66	0.76	0.58
Mental Performance Capability	0.49	0.58	0.65	0.63
Emotional Balance	0.46	0.56	0.60	0.45
Overall Recovery	0.64	0.66	0.72	0.65

Muscular Stress	0.69	0.65	0.68	0.55
Lack of Activation	0.74	0.46	0.63	0.52
Negative Emotional State	0.70	0.56	0.62	0.52
Overall Stress	0.67	0.65	0.72	0.63

*ARSS, Acute Recovery and Stress Scale; SRSS, Short Recovery and Stress Scale; ^*a*^, reference values as published in the German manual (Kellmann et al., 2016, p. 63); all correlations are significant on the level *p* < 0.001.*

The results of the CFA and the Multigroup CFA between adults and adolescents (phase 1) with the original ARSS are depicted in Table 7. Both groups revealed decent fit indices in the *Recovery* dimension. In addition, all of the fit indices were within the recommended thresholds in the three conditions of invariance analysis. The CFI did not change when comparing models of configural and metric invariance, while the change of the remaining fit indices did not exceed the suggested cut-off values. Regarding the *Stress* dimension, the initial model was acceptable despite the RMSEA values among adults (χ^2^ = 400.80, *df* = 98, *p* < 0.001, CFI = 0.914, SRMR = 0.071, RMSEA = 0.091 [90%-CI = 0.082,0.101]). Model fit was slightly improved following modifications (i.e., covariation of measurement errors within *Lack of Activation*) which were then applied to the model of the adolescents who showed a better fit than the adults (Table 7). The analyses of measurement invariance showed good fits, despite the borderline RMSEA’s upper limit of the 90%-CI in each step. The change of the fit indices was within the recommended thresholds, while the CFI increased by 0.001 in the model of metric invariance.

**TABLE 7:** Multigroup confirmatory factor analysis with the adult sample (*n* = 442) and adolescents of phase 1 (*n* = 199).

	**Model**	**χ2**	***df***	***p***	**CFI**	**SRMR**	**RMSEA**	**90% CI**		**ΔCFI**	**ΔSRMR**	**ΔRMSEA**
Recovery Dimension	Adults	253.37	98	<0.001	0.944	0.047	0.067	0.057	0.077	−/−	−/−	−/−
	Adolescents (Phase 1)	152.57	98	<0.001	0.950	0.054	0.058	0.039	0.075	−/−	−/−	−/−
	Configural Invariance	408.58	196	<0.001	0.950	0.047	0.064	0.056	0.073	−/−	−/−	−/−
	Metric Invariance	421.97	208	<0.001	0.950	0.049	0.062	0.054	0.071	0.000	0.002	−0.002
	Scalar Invariance	473.28	220	<0.001	0.941	0.056	0.066	0.058	0.074	−0.009	0.007	0.004

Stress Dimension	Adults	321.82	94	<0.001	0.935	0.063	0.081	0.072	0.091	−/−	−/−	−/−
	Adolescents (Phase 1)	164.19	94	<0.001	0.930	0.063	0.070	0.051	0.087	−/−	−/−	−/−
	Configural Invariance	480.52	188	<0.001	0.934	0.059	0.078	0.069	0.086	−/−	−/−	−/−
	Metric Invariance	473.33	200	<0.001	0.935	0.065	0.075	0.066	0.084	0.001	0.006	−0.003
	Scalar Invariance	525.59	212	<0.001	0.925	0.073	0.078	0.070	0.086	−0.010	0.008	0.003

*CFI, Comparative Fit Index; SRMR, Standardized Root Mean Residual; RMSEA, Root Mean Error of Approximation; CI, Confidence Interval; specifications of the final Stress model are identical between groups.*

The results of the CFA and Multigroup CFA among both groups of phase 2 are displayed in Table 8. The initial *Recovery* model fit was acceptable despite the RMSEA values for the adolescents (χ^2^ = 221.69, *df* = 98, *p* < 0.001, CFI = 0.926, SRMR = 0.054, RMSEA = 0.077 [90%-CI = 0.063,0.090]), while it was overall somewhat poor for the children (χ^2^ = 165.54, *df* = 98, *p* < 0.001, CFI = 0.862, SRMR = 0.082, RMSEA = 0.085 [90%-CI = 0.062,0.106]). Table 8 shows the fit indices of the final model. Measurement invariance was found in each step, while the upper limit of the 90%-CI of RMSEA slightly exceeded the recommended threshold in each of the models. The modified *Stress* model of the adult sample was applied to both groups of phase 2. While the fit indices were just within an acceptable range for the adolescents (χ^2^ = 178.98, *df* = 94, *p* < 0.001, CFI = 0.947, SRMR = 0.058, RMSEA = 0.069 [90%-CI = 0.053,0.084]), it was considerably poorer among the children (χ^2^ = 191.48, *df* = 94, *p* < 0.001, CFI = 0.850, SRMR = 0.082, RMSEA = 0.106 [90%-CI = 0.084,0.127]). A second modification through covariance relationships within *Muscular Stress* led only to marginal improvements of the model in both groups (see Table 8). Nevertheless, measurement invariance was found with acceptable fit indices and changes of fit, despite the RMSEA’s upper limit in each model.

**TABLE 8:** Multigroup confirmatory factor analysis among phase 2 participants with adolescents (*n* = 261) and children (*n* = 115).

	**Model**	**χ2**	***df***	***p***	**CFI**	**SRMR**	**RMSEA**	**90% CI**		**ΔCFI**	**ΔSRMR**	**ΔRMSEA**
Recovery Dimension	Adolescents (Phase 2)	198.53	94	<0.001	0.938	0.051	0.072	0.058	0.086	−/−	−/−	−/−
	Children	158.71	94	<0.001	0.870	0.078	0.084	0.061	0.106	−/−	−/−	−/−
	Configural Invariance	357.78	188	<0.001	0.923	0.056	0.076	0.064	0.088	−/−	−/−	−/−
	Metric Invariance	368.82	200	<0.001	0.923	0.061	0.073	0.061	0.085	0.000	0.005	−0.003
	Scalar Invariance	387.42	212	<0.001	0.921	0.062	0.072	0.061	0.083	−0.002	0.001	−0.001

Stress Dimension	Adolescents (Phase 2)	166.44	91	<0.001	0.952	0.055	0.066	0.050	0.082	−/−	−/−	−/−
	Children	174.90	91	<0.001	0.870	0.082	0.100	0.078	0.123	−/−	−/−	−/−
	Configural Invariance	335.23	182	<0.001	0.931	0.060	0.077	0.064	0.090	−/−	−/−	−/−
	Metric Invariance	351.77	194	<0.001	0.930	0.066	0.076	0.063	0.088	−0.001	0.006	−0.001
	Scalar Invariance	371.50	206	<0.001	0.928	0.067	0.075	0.062	0.087	−0.002	0.001	−0.001

*CFI, Comparative Fit Index; SRMR, Standardized Root Mean Residual; RMSEA, Root Mean Error of Approximation; CI, Confidence Interval; specifications of the final Stress model are identical between groups.*

## Discussion

In the light of early specialization and intensified training among developing athletes, monitoring training load and the recovery-stress state has gained significance in youth sport as part of effective training management and health prevention. As it is questionable whether self-report measures which were developed for and with adults can be applied among younger athletes, it was the aim of the present study to examine psychometric properties of two established questionnaires in their original form as well as with initial modifications to approach the level of comprehension.

Overall, the results confirm that the understanding of the items is difficult among younger athletes. Although issues other than the lack of comprehensibility may be responsible for missing data, the majority of the children did not return complete ARSS ratings and most of the missing values were due to the “*I don’t understand*” option. Specifically, the age group of 10- and 11-year-olds was identified to most frequently mark items as “*I don’t understand*” across the dimensions of *Recovery* and *Stress*, with at least one item of *Mental Performance Capability*, *Muscular Stress*, *Lack of Activation*, and *Negative Emotional State*. The descriptive statistics of the items served as another indicator of limited applicability as recovery items were consistently rated higher and stress items lower by the participants of the study groups compared to the validation sample. One reason could be that the younger athletes have either not yet developed the awareness and interpretation of their psychophysiological state or they have difficulties in expressing their current perception of recovery and stress in numerical graduations. This may explain the low internal consistency of the ARSS scale *Emotional Balance* and the low item-total correlation of item 2 (which corresponds to “feeling down”) among the children group in phase 2, although a description of that item was provided. Another explanation may be the number of response options. Borgers et al. (2004) found out that offering more than six options appeared to cause a decrease in scale reliability for children between 8 and 16 years.

Interestingly, modifying single items of the ARSS seemed to contribute to a better understanding among the adolescents, as improved Cronbach’s α values were found comparing phase 1 to phase 2. In general, it is recommended that the instructions and questions of a questionnaire should be simple with clear and unambiguous wording. This is especially important when working with children between 8 and 11 years (Borgers et al., 2000). As the ARSS only presents a list of adjectives, which may partly have ambiguous meanings, limited applicability seems to be induced among the children group and response bias may be an issue. Borgers et al. (2003) argue that children younger than 10 years might not be able to answer questionnaires reliably, which is expressed in their difficulties to apply the response options. Moreover, it seems that adolescents around the age of 11 may provide consistent answers which improves with age and may be stabilized around the age of 14 (Borgers et al., 2000).

The descriptive item statistics of the SRSS were comparable across the study groups, although the stress ratings were lower than in the validation sample. While the original SRSS revealed acceptable internal consistency among adolescents, which was quite similar to the validation sample, the modified SRSS indicated even higher values for adolescents as well as children. It has to be noted that the missing option to mark “*I don’t understand*” is a limiting factor of the study design, and the issue of response bias cannot be ruled out. Nevertheless, the results suggest that the SRSS might be applicable for athletes from the age of 10 onward. The correlational patterns of the ARSS and the SRSS across the study groups imply that both assess the recovery-stress state, but they can be considered as independent questionnaires, as the coefficients did not reveal perfect correlations. This finding was also present across different data collections with the original tools (Kellmann et al., 2016; Nässi et al., 2017a; Kellmann and Kölling, 2019).

Multigroup CFA was performed to examine if the ARSS is measuring the same construct across groups. As a first step, the models need to show a decent model fit in each group separately (Cheung and Rensvold, 2002). This was found for the *Recovery* model in every group. Considering the rather borderline values of the RMSEA’s 90%-CI across groups, the *Stress* construct might be critically discussed. Especially among the children, the model seems to fit somewhat poorly to the data. However, the descriptive rather than normative nature of the fit indices and their cut-offs has to be pointed out, so that there is actually no consensus definition of an ideal fit (Worthington and Whittaker, 2006). At the level of configural invariance, the models of the adults and adolescents of phase 1 as well as those of phase 2 were combined. In both group comparisons, the model fit indicates that the basic factor structure can be considered equal among the groups. Thus, the original items of the ARSS seem to assess the same pattern of *Recovery* and *Stress* of participants between 14 and 16 years as of adults. The same conclusion can be drawn for the modified ARSS. Weak measurement invariance can be assumed when the factor loadings are equivalent between groups. The model fit did not decrease out of the recommended range in either condition (i.e., original ARSS, modified ARSS) nor in the dimensions (i.e., *Recovery*, *Stress*). Even the third model seems to provide acceptable fit which indicates strong measurement invariance that would allow for the comparison of the latent mean between groups. Nevertheless, in the present study, data were collected in a range of naturalistic situations which could not be controlled. As the underlying construct of acute recovery and stress represent a state that is assumed to change over the course of time (and in response to stress or recovery stimuli), the within-individual stability of the construct needs to be analyzed over time.

Considering the results and initial implications, coaches and practitioners need to appreciate that the period of adolescence is critical for the maturation of neurobiological processes, among others, which may contribute to cognitive and affective behavior (Yurgelun-Todd, 2007). Moreover, Blakemore and Choudhury (2006) point out the sensitivity of the brain to experiential input in terms of executive function and social cognition due to the synaptic reorganization. The developing brain as well as behavioral and cognitive systems mature along different timetables which causes heightened vulnerability in adolescents (Steinberg, 2005). In terms of cognitive efficiency in response to emotionally related stimuli, McGivern et al. (2002) found a decrement at the onset of puberty. This may support the rather poor statistics of the emotionally related scales in the present study. While it may be possible in surveys to use standardized questionnaires that are similar to those for adults among the age group of 11 to 15–16 years (Borgers et al., 2000), precautions should be considered. As the present study revealed, it is important to test the questionnaires among the target populations and provide modifications to enhance reliable responses. In some cases, it may be sufficient to explain the questionnaire when handed out for the first time and to be available for further questions. Otherwise, items or scales that have been known as being problematic should rather not be interpreted and analyzed at all.

### Limitations and Future Directions

Some limitations of the study, especially regarding phase 1, need to be commented on. As the anonymity of the athletes in phase 1 was the priority, valuable information could not be assessed and the analyses were limited to the overall group level. Moreover, pre- post measurements to examine improvements of understanding within the individuals were not possible. On the other hand, the high performance level of the phase 1 group was an advantage, as the participants were familiar with training and exercise which may facilitate their general understanding of the topic of the questionnaires.

Furthermore, it was the aim to explore the psychometric properties among those who provided complete responses which caused a considerable reduction of the sample sizes. Appropriate statistical measures, such as multiple imputation, may be considered in future analyses to adjust for missing item scores. Although it may be of minor relevance at the level of the items’ understanding, team sport athletes were somewhat overrepresented. Therefore, the present results should be considered as preliminary investigation in this area. Moreover, it seems worthwhile to analyze the psychometric properties for each age group to identify possible cut-offs which differentiate between the applicability of the original and the need for modified versions. Therefore, larger sample sizes should be recruited in future studies. This may further allow for separate gender analyses, since female athletes were underrepresented in this study. As the participants gave their responses at different times and various settings (e.g., in a training camp, before or after an intensive training), the sensitivity to change needs to be investigated systematically once the modifications are completed.

In the present study, a top-down approach was chosen to evaluate the recovery-stress model that was established for adults among the younger clientele. As suggested by Ravens-Sieberer and Bullinger (1998), a mixture of top-down and bottom-up methods is preferable. With the help of bottom-up tactics, the children’s concepts of recovery and stress and perceptions of their psychophysiological response to training as well as relevant recovery and stress dimensions may be considered.

## Ethics Statement

This study was carried out in accordance with the recommendations of the ethical committee of the Faculty of Psychology at the Ruhr University Bochum with written informed consent from all subjects. All subjects gave written informed consent in accordance with the Declaration of Helsinki. The protocol was approved by the ethical committee of the Faculty of Psychology at the Ruhr University Bochum (application number 308).

## Author Contributions

SK planned and designed the study, conducted measurements, analyzed the data, and prepared the manuscript. AF, TM, and MP edited the manuscript. MK planned and designed the study and edited the manuscript. All authors read and approved the submitted version.

## Conflict of Interest

The authors declare that the research was conducted in the absence of any commercial or financial relationships that could be construed as a potential conflict of interest.
